# Regional variations and temporal trends of atopic diseases in Germany

**DOI:** 10.1111/ddg.15926

**Published:** 2026-01-16

**Authors:** Marie Sander, Matthias Augustin, Valerie Andrees, Sandra Hischke, Jobst Augustin

**Affiliations:** ^1^ Institute for Health Services Research in Dermatology and Nursing (IVDP) University Medical Center Hamburg‐Eppendorf (UKE) Hamburg Germany

**Keywords:** Atopic dermatitis, bronchial asthma, epidemiology, geography, rhinoconjunctivitis

## Abstract

**Background and Objectives:**

Prevalence rates of atopic diseases (atopic dermatitis [AD], allergic asthma [AA], and allergic rhinoconjunctivitis [ARC]) have increased in Germany. However, there is a lack of simultaneous consideration and of precise data on the spatiotemporal variation of these diseases. This study investigates the spatiotemporal variation of atopic diseases in Germany.

**Methods:**

Analyses based on outpatient care data (2011‐2020) of all statutory health insured persons in Germany. Spatiotemporal analyses of prevalence rates were performed on county levels and statistical smoothing were applied.

**Results:**

Prevalence rates increased for all three diseases. Moderate but significant (p < 0.05) correlations were among others observed between ARC and AA (r = 0.47).

Correlations with the east‐west gradient were significant for all diseases, meaning higher prevalence rates of AD in the east and higher prevalence rates of AA and ARC in the west. The correlation coefficients showed significant values between the north‐south gradient and AD, as well as between the north‐south gradient and AA, implying higher prevalences in northern Germany.

**Conclusions:**

Prevalence rates for atopic diseases have increased over the past decade. Regional patterns are different for AD and the other atopic diseases. The reasons for the increase and the variations need to be analysed in detail with a view to improving prevention and health care.

## INTRODUCTION

Atopic diseases have become more prevalent in recent decades and are part of everyday life for about 20% of the world's population.^1^ Atopy is a genetic predisposition which includes the tendency for elevated immunoglobulin E (IgE) antibodies, dysregulations in the adaptive and innate immune system, and disruptions of skin barrier function.^1^, ^2^, ^3^ Atopic diseases, including atopic dermatitis (AD), allergic rhinoconjunctivitis (ARC), and allergic asthma (AA) develop differently over the course of a lifetime. While some become more severe, others may subside. Typically, AD occurs first in life, followed by allergic asthma and allergic rhinitis.^1^, ^4^, ^5^ However, the phases of each condition may change or overlap and recur at any point in life.

Atopic dermatitis is a chronic inflammatory skin disease with multiple forms of manifestation. It usually affects young children (10%–20% lifetime prevalence), but adults may also be affected (1%–3% lifetime prevalence).^1^, ^6^ The lifetime prevalence for adults between 18 and 79 years in Germany is 3.4%.^7^ In industrialized countries, the prevalence of AD increases more than in countries with more rural areas. The prevalence also varies among groups of people with similar genetic backgrounds, so environmental influences seem to be an important factor.^1^

Bronchial asthma is also a chronic inflammatory and obstructive lung disease.^8^ It can be expressed as extrinsic (allergic) asthma, intrinsic (non‐allergic) asthma, or a mixed form, affecting about a quarter of a billion people worldwide. The lifetime prevalence is 16.5% in adults between 18 and 79 years in Germany.^7^

Rhinoconjunctivitis manifests itself primarily in the swelling of mucous membranes in the mouth and nose area, rhinitis, itching, and watery eyes. Children and adolescents are particularly affected.^9^ In Germany, the lifetime prevalence of ARC of 14.8% in adults between 18 and 79 years.^7^

The three diseases are thus closely related and may occur simultaneously or sequentially in an individual. Therefore, a joint regional assessment of prevalence rates is useful. Although atopic diseases and comorbidities have frequently been the subject of publications, there is a lack of data on the regional spread in Germany. In particular, simultaneous analyses of AD, AA, and ARC for regional characteristics at the county level have been lacking.

To gain new insights into the characteristics of atopic diseases and to add value for care, this study *(1)* examines the regional prevalence rates of AD, AA, and ARC in Germany at the county level and *(2)* describes the change in their prevalence rates over time between 2011 and 2020. In addition, geographical patterns of disease prevalence are analyzed for spatial associations among the diseases themselves and in relation to north–south and east–west gradients. This is because a marked north–south gradient has also been shown in psoriasis (e.g. Andrees et al.^10^), and it is unclear whether this is also present in the diseases investigated here.

## MATERIALS AND METHODS

### Dataset and data preparation

The dataset for this ecological secondary data study was provided by the *National Association of Statutory Health Insurance Physicians* (KBV) and is available at the level of counties (n = 401) (Nomenclature des Unités territoriales statistiques – NUTS 3) for the years 2011 to 2020. All individuals covered by one of the currently 97 statutory health insurance (SHI) funds in Germany are represented, corresponding to approximately 90% (70.2 million) of the German population. This data set includes data solely for treatments covered by the SHI.

Since chronic diseases can have mild to severe courses and as the milder courses were also to be considered in this study, we defined cases as at least one billed, confirmed diagnosis of AD, AA, or ARC in one quarter within a twelve‐month time‐period (M1Q definition). Individuals older than 90 years or with ambiguous gender information were excluded from the dataset. The regional assignment of cases was based on the place of residence. Under these conditions, the dataset contains information on the number of people diagnosed with AD, AA, or ARC (Table 1).

**TABLE 1:** Overview of the dataset.

Primary indication	ICD‐10	Description
Atopic dermatitis (AD)	L20.0	Prurigo Besnier
	L20.8	Other atopic (endogenous) eczema
	L20.9	Atopic (endogenous) eczema, unspecified
Allergic asthma (AA)	J45.0	Predominantly allergic asthma
	J45.8	Mixed forms of bronchial asthma
	J45.9	Bronchial asthma, unspecified
Allergic rhinoconjunctivitis (ARC)	J30.1	Allergic rhinopathy due to pollen, hay fever, pollen allergy otherwise not specified Pollinosis
	J30.2	Other seasonal allergic rhinopathy
	J30.3	Other allergic rhinopathy All‐round allergic rhinopathy
	J30.4	Allergic rhinopathy, unspecified

To calculate the relative frequencies for the 401 counties (territory status 2020) in Germany, the absolute number of cases per county was divided by the absolute number of people with statutory health insurance per county. The number of people with statutory health insurance is available for 2020 and was used to calculate the prevalence rates for all years.

### Descriptive and temporal trend analyses

Annual prevalence rates per 100,000 people with statutory health insurance for 2011 to 2020 were calculated for the 401 counties in Germany. In addition, for all three diseases, the mean prevalence rates with standard deviation as well as minimum and maximum values were determined. The Gini coefficients (GC) were calculated to determine the equality or inequality of the distributions of the diseases across the counties of Germany. The Gini coefficient varies between 0 and 1, where 0 corresponds to a completely equal distribution, with an increasingly uneven distribution the closer the value is to 1.^11^ We assessed the prevalence change between 2010 and 2020 for each disease.

### Spatial analyses

Pearson correlation coefficients with corresponding 95% confidence intervals (CI) were calculated to determine associations between the different atopic diseases. The analysis according to Pearson examines linear relationships between continuous variables. It ranges from –1, representing total negative linear correlation, to 1, representing total positive correlation. A value of 0 indicates no correlation.

In addition, the spatial distributions of the prevalence rates for the year 2020 were examined. We detected high‐rate counties by calculating the 75% percentile for each disease and considered those counties with prevalence rates above the respective 75% percentile. In this manner, counties with high rates for one, two or all three diseases were identified.

The prevalence rates for 2020 were smoothed using an exploratory Bayesian method with 1,000 sequential Monte Carlo iterations to determine the deviations of the actual values from the expected values in each county. As an expected value, we defined the mean value of the German prevalence rate for the respective disease. This model assumes that spatially close counties have more similar values than more distant ones. We use a binary adjacency matrix to consider the direct neighbors of a county. In Bayesian smoothing, 1 represents the expected value of a county, meaning that counties with values below 1 have lower prevalence rates than expected and counties above 1 have higher prevalence rates than expected.^12^, ^13^

Global Moran's I was calculated to test the data for spatial autocorrelation, that is, to examine, how closely values are spatially clustered. The Global Moran's index varies from –1 to 1 and, with a significant p value, indicates that there is spatial autocorrelation. Values above 0 indicate high similarity within the neighborhood, while values below 0 indicate high dissimilarity. Values equal to 0 indicate no spatial autocorrelation.^14^, ^15^

For the detection of associations with celestial directions, scatter plots with associated linear regression line and 95% CI band and Pearson correlations and 95% CI of the disease prevalence rates with latitude for north‐south gradients, and longitude for east‐west gradients, were carried out. To execute the analyses on longitude and latitude, centroid points were formed for each county, with the corresponding information on prevalence rates and the exact longitude and latitude.

A p value of < 0.05 was defined as statistically significant.^16^ For statistical and spatial data analyses QGIS 3.22.1‐Białowieża (QGIS Development Team) and RStudio 2022.02.1+461 “Prairie Trillium” (RStudio, PBC, Boston, MS, USA) were used.

## RESULTS

### Descriptive and temporal trend analyses

Prevalence rates for all three diseases increased from 2011 to 2020 in Germany: The annual prevalence of atopic dermatitis increased from 3,929 to 4,091 per 100,000, for AA it increased from 5,985 to 7,087 per 100,000 and for ARC from 6,525 in 2011 to 7,819 per 100,000 in 2020 (Table 2).

**TABLE 2:** Annual prevalence rates at the county level per 100,000 persons with statutory health insurance.

	2011	2012	2013	2014	2015	2016	2017	2018	2019	2020	Change
*Atopic dermatitis*											
Mean	3,929.3	3,900.8	3,968.6	4,053.6	4,088.9	4,111.2	4,109.4	4,127.7	4,105.9	4,091.7	+ 162.4
Median	3,799.2	3,781.6	3,839.2	3,923.2	3,977.0	3,991.8	4,001.5	4,029.5	4,032.5	3,996.7	
Standard deviation	888.7	864.3	904.4	867.0	886.6	850.4	868.9	833.6	818.2	808.2	
Min	2,092	2,147	2,207	2,278	2,258	2,369	2,346	2,296	2,354	2,337	
Max	7,685	7,668	7,786	8,001	7,883	7,606	7,512	7,116	7,021	6,777	
Gini Coefficient	0.121	0.117	0.116	0.115	0.114	0.112	0.111	0.110	0.109	0.108	
Moran's I	0.446^*^	0.455^*^	0.414^*^	0.462^*^	0.443^*^	0.470^*^	0.446^*^	0.472^*^	0.464^*^	0.467^*^	
*Allergic asthma*											
Mean	5,985.0	6,015.2	6,311.9	6,518.0	6,640.4	6,796.1	6,831.2	6,999.3	7,075.8	7,087.4	+ 1,102.3
Median	5,955.9	5,984.4	6,316.1	6,468.2	6,586.2	6,758.5	6,836.9	6,948.0	7,071.9	7,062.9	
Standard deviation	1,056.4	1,073.0	1,123.3	1,135.6	1,157.9	1,183.8	1,180.8	1,207.9	1,184.7	1,137.9	
Min	3,485	3,403	3,719	3,626	3,641	3,785	3,766	3,831	3,835	3,844	
Max	9,069	9,159	9,206	9,454	9,652	9,863	10,090	10,883	10,384	10,523	
Gini Coefficient	0.099	0.100	0.100	0.098	0.098	0.098	0.097	0.097	0.094	0.090	
Moran's I	0.578^*^	0.571^*^	0.575^*^	0.567^*^	0.574^*^	0.582^*^	0.570^*^	0.574^*^	0.551^*^	0.526^*^	
*Allergic rhinoconjunctivitis*											
Mean	6,525.8	6,396.6	6,803.6	7,086.4	7,203.9	7,443.9	7,355.9	7,656.9	7,732.5	7,819.7	+ 1293.9
Median	6,433.6	6,314.8	6,737.5	7,073.6	7,200.5	7,422.0	7,402.3	7,622.7	7,692.5	7,825.5	
Standard deviation	932.6	895.5	955.7	964.5	975.9	1,029.5	1,010.2	1,065.5	1,050.0	1,051.5	
Min	4,161	4,040	4,576	4,704	4,821	4,877	4,699	4,764	4,507	4,962	
Max	10,215	10,018	10,264	9,944	10,122	10,550	10,114	10,879	10,966	11,203	
Gini Coefficient	0.080	0.078	0.079	0.076	0.076	0.078	0.077	0.078	0.076	0.075	
Moran's I	0.530^*^	0.471^*^	0.479^*^	0.454^*^	0.459^*^	0.457^*^	0.432^*^	0.469^*^	0.416^*^	0.395^*^	

* Statistically significant (p value < 0.05)

The Gini coefficients are similar for all three diseases and decreased slightly from 2011 to 2020. For AD, the GC was highest at 0.121 in 2011 and lowest in 2020 with 0.108. The GC for AA showed the highest value in 2012 with 0.10 and the lowest in 2020 with 0.09. For ARC, the GC from all three diseases was closest to 0 and was highest at 0.08 in 2011 and lowest in 2020 with 0.075 (Table 2).

**FIGURE 1:**
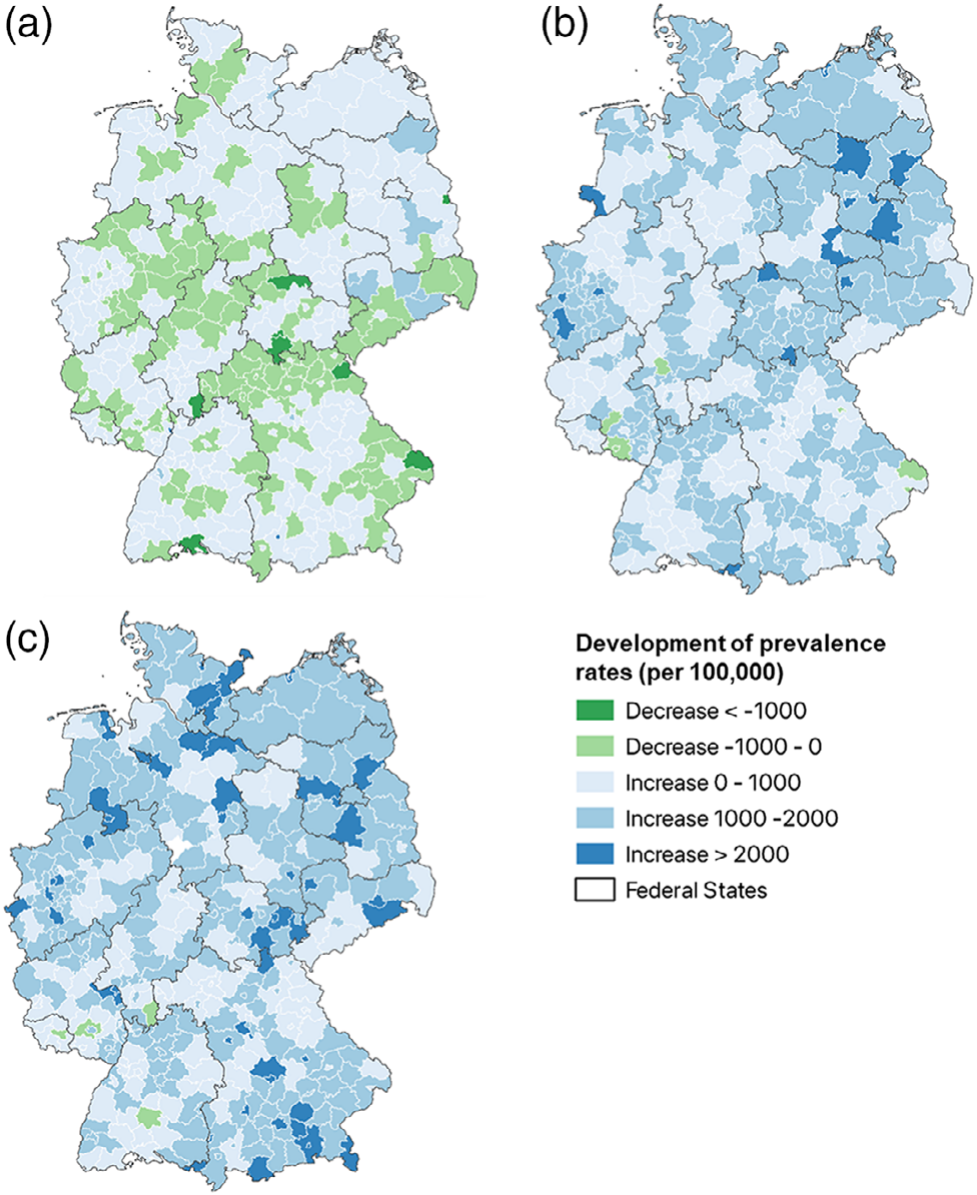
Development of prevalence rates (per 100,000 persons with statutory health insurance) from 2011 to 2020 in Germany: (a) atopic dermatitis, (b) allergic asthma, (c) rhinoconjunctivitis.

In more than half of the counties (n = 266), the prevalence rate of AD increased from 2011 to 2020, but in 135 counties there was also a significant decrease. A strong increase in prevalence was found in north‐east Germany in particular. For AA and ARC, nearly all counties (n = 393) show an increase (Figure 1).

### Spatial analyses

With respect to the prevalence at county level, the Pearson correlation coefficients between ARC and AA range between 0.40 and 0.47 (p < 0.05) in the years 2011 to 2020. For AD and AA, the correlation coefficients range from 0.16 to 0.22 (p < 0.05). The correlation between AD and ARC was not significant (Table 3).

**TABLE 3:** Pearson correlation coefficients between atopic diseases.

	2011	2012	2013	2014	2015	2016	2017	2018	2019	2020
***Atopic dermatitis – allergic asthma***										
**r**	0.16^*^ [0.06,0.25]	0.22^*^ [0.12,0.31]	0.21^*^ [0.11,0.30]	0.18^*^ [0.09,0.28]	0.18^*^ [0.09,0.28]	0.20^*^ [0.10,0.29]	0.20^*^ [0.11,0.30]	0.19^*^ [0.10,0.29]	0.18^*^ [0.09,0.28]	0.16^*^ [0.07,0.26]
***Atopic dermatitis – rhinoconjunctivitis***										
**r**	0.09 [‐0.01,0.19]	0.13^*^ [0.03,0.23]	0.07 [‐0.02,0.17]	0.06 [‐0.04,0.16]	0.05 [‐0.04,0.16]	0.06 [‐0.03,0.16]	0.08 [‐0.01,0.18]	0.08 [‐0.01,0.18]	0.11^*^ [0.01,0,21]	0.09 [‐0.01,0.19]
***Rhinoconjunctivitis – allergic asthma***										
**r**	0.43^*^ [0.34,0.50]	0.42^*^ [0.33,0.50]	0.42^*^ [0,34,0,50]	0.41^*^ [0.33,0.49]	0.41^*^ [0.32,0.49]	0.43^*^ [0.35,0.51]	0.43^*^ [0.35,0.51]	0.47^*^ [0.39,0.54]	0.44^*^ [0.36,0.52]	0.40^*^ [0.32,0.48]

* Statistically significant (p value < 0.05)

**FIGURE 2:**
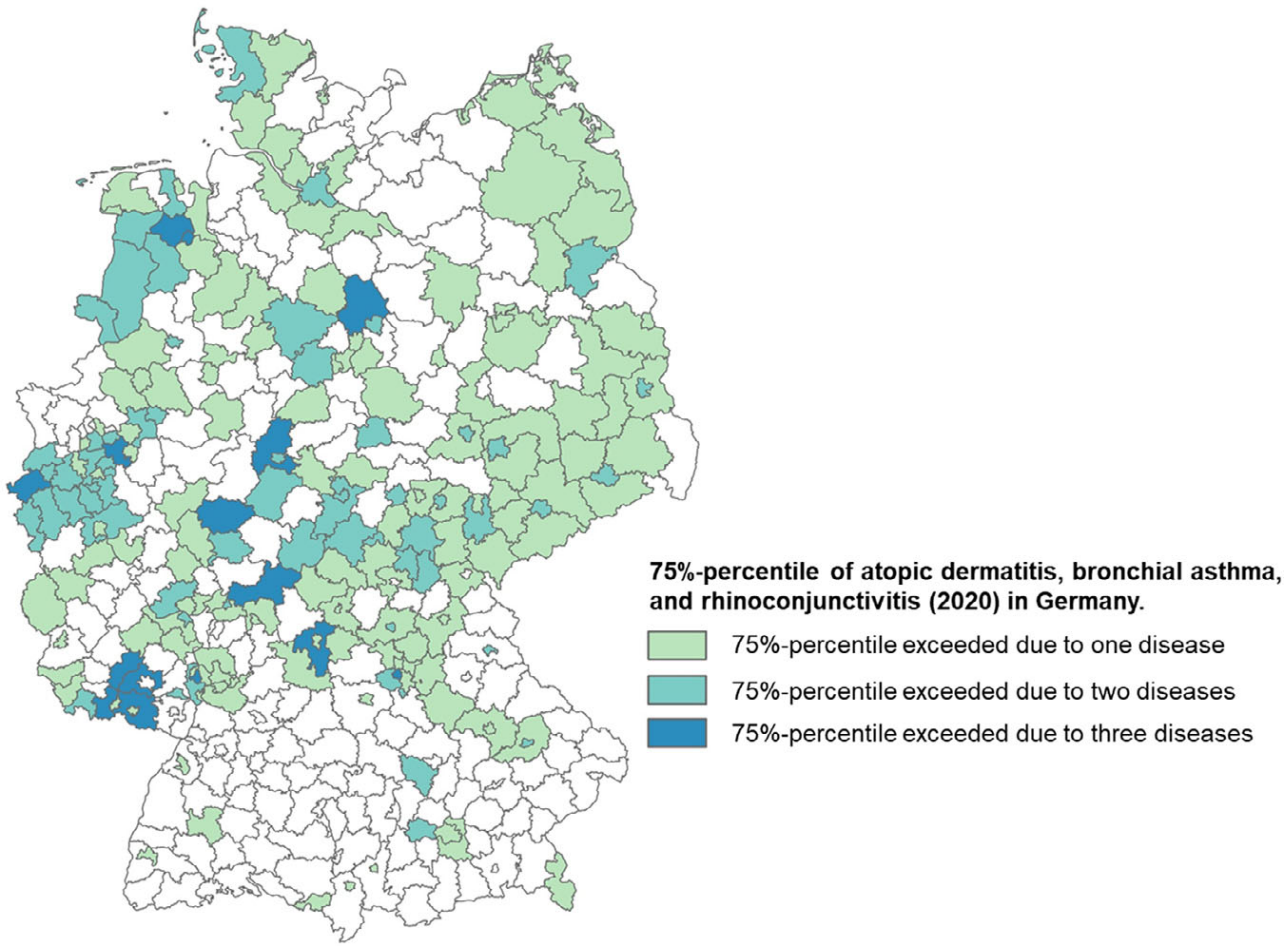
75th percentile of atopic dermatitis, allergic asthma, and rhinoconjunctivitis (2020) in Germany.

The distribution of diseases with a prevalence rate above the 75th percentile in the respective county shows many counties with a high prevalence rate of AD in eastern Germany. Two diseases with prevalence rates above the 75th percentile are particularly evident in western German counties and in the north. In addition, prevalence rates above the 75th percentile for all three diseases occur in 16 counties in central and western Germany (Figure 2).

**FIGURE 3:**
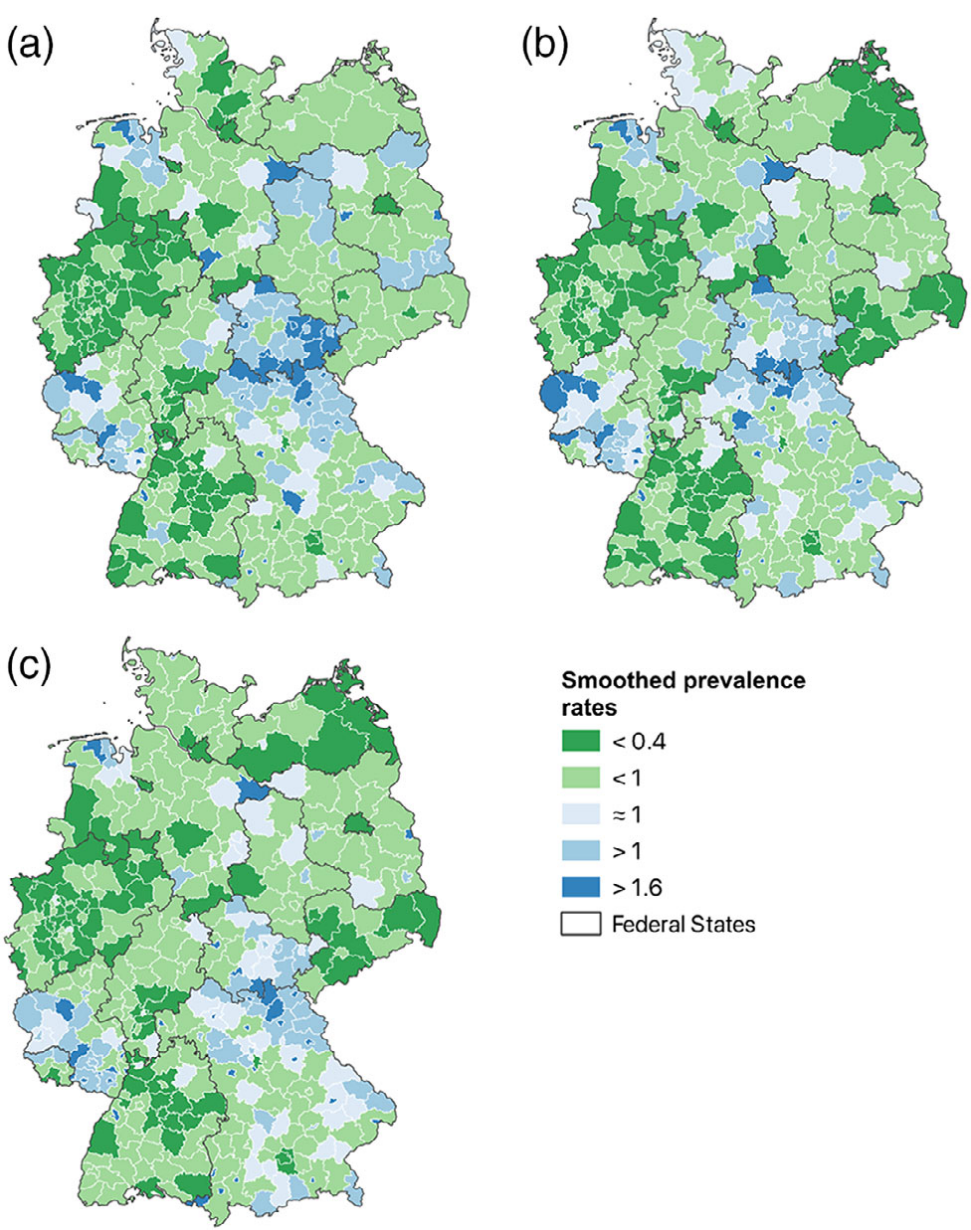
Smoothed prevalence values for German counties based on Bayesian modeling: (a) atopic dermatitis, (b) allergic asthma, (c) rhinoconjunctivitis.

Bayesian smoothing revealed similar patterns, particularly for ARC and AA, with slightly greater deviations for AD. The smoothed values ranged from 0.03 for AA in Berlin, indicating a lower prevalence than expected, to 4.12 for AD in Pirmasens (Rhineland‐Palatinate), indicating a markedly higher prevalence than expected. Higher values than expected for all three diseases are found in northern Bavaria and Thuringia, as well as in western Rhineland‐Palatinate. Lower values than expected are rather heterogeneously distributed and are especially found for AD in western North Rhine‐Westphalia and Lower Saxony, as well as in Baden‐Württemberg. For AA and ARC, low values are found in North Rhine‐Westphalia, Lower Saxony and Baden‐Württemberg, but also to some extent in Mecklenburg‐Western Pomerania, Berlin and Saxony (Figure 3).

The global Moran's I indicates spatial autocorrelation, with positive significant values between 0.395 for ARC and 0.582 for AA for all three conditions.

In the correlation analysis of the three diseases with longitude and latitude, AD shows a correlation coefficient of r = 0.28 (95% CI 0.18–0.37) with longitude and r = 0.28 (95% CI 0.19–0.37) (p < 0.05) with latitude (online supplementary Appendix S1): The further east and the further north in Germany, the higher the prevalence of AD. For AA as well, statistically significant correlation coefficients with longitude and latitude were found with r = –0.36 (95% CI –0.44 to –0.27) and r = 0.31 (95% CI 0.22 to 0.40]), respectively, indicating that the further east in Germany, the lower, and the further north in Germany, the higher the prevalence of AA. For ARC, a statistically significant correlation with longitude was found (r = –0.20; 95% CI –0.29 to –0.10), whereas no statistically significant correlation with latitude was observed (r = –0.05; 95% CI –0.15 to 0.05) (online supplementary Table S1).

## DISCUSSION

The objective of this spatiotemporal analysis of atopic diseases in Germany was to identify potential differences in annual prevalences at county level. The data demonstrate significant regional disparities of AD, AA, and ARC. Furthermore, an increasing total prevalence was detected for all three diseases in the past decade, particularly for AA and ARC. Both the mean prevalence rate in Germany and the increase in prevalence from 2011 to 2020 were highest for ARC. It is striking that the developmental trend in the prevalences of ARC and AA were very similar, while the increase in AD was significantly lower. For the latter, many counties even show a slight decline in prevalence rates.

Primary and secondary data from studies on smaller German populations show partly comparable prevalence rates for AD and AA.^7^, ^17^ The twelve‐month prevalence of 18‐ to 79‐year‐old study participants in the *German Health Interview and Examination Survey for Adults* (DEGS), a primary data collection from 2011, was 2.2% for AD and 5.0% for AA, which is lower than the estimated prevalence rates in our study (in 2011 3.9% for AD and 6.0% for AA).^7^ By contrast, the prevalence of ARC in DEGS (12.0%) was significantly higher than in our study (6.5%).^7^ One reason for the difference could be that people with mild rhinoconjunctivitis do not regularly consult a physician and therefore are not identifiable in our dataset. Another reason could be an underestimation of the rate as we only had the number of insured persons from 2020 and thus overestimated the denominator for the prevalence estimate.

Although the GCs do not show large inequalities in the distribution of prevalence rates, the correlation analyses with longitude and latitude reveal differences in the distribution. These differences become clearer when viewing the 75% percentile of the three diseases. High prevalence rates of AD occur mainly in East Germany and show only weak or no significant correlations with the other two diseases, whereas the high prevalences of AA and ARC cluster in the center of Germany, in the west and in the north. This is also supported by the significant correlation coefficients above r = 0.4 between the two diseases. Although the association between atopic diseases is well established, there are no studies that explicitly examine the association between the three diseases in the regions with the highest prevalence rates. Other studies have found a strong association between AA and AD, which is not so strongly reflected in our results.^8^ In contrast, the association of AD and AA with ARC, as described in other studies, was also detected here.^18^, ^19^

As shown by Engebretsen et al., climatic conditions can influence atopic diseases. Distance from the equator correlates with the prevalence of skin diseases such as AD.^20^ Augustin et al. have also demonstrated an association between latitude and psoriasis.^2^ Low humidity, low UV exposure, different distribution of daylight, and low temperatures in northern areas could lead to higher prevalence rates of AD.^20^ Links to distance from the equator have also been postulated for AA and ARC. While asthma shows positive correlations with increased CO_2_, temperature and pollen count, high mean temperatures and low humidity were also related to higher prevalence rates of ARC.^17^, ^21^ These findings are partly supported by the present study, which shows significantly higher prevalences in northern counties, especially for AD and AA. As Germany is geographically a rather small country with an extension of about 900 km from north to south and 600 km from west to east, associations with climatic factors and prevalence rates of the three diseases should be treated with caution. Other socio‐economic and infrastructural factors may play a more important role.

Less frequently studied is the association of atopic diseases with longitude, i.e., the differences between eastern and western regions which are affected by the historical development in Germany. At the time of the reunification (1990) of the Federal Republic in the west and the German Democratic Republic in the east, eastern German areas had higher prevalence rates for many diseases.^22^ However, the opposite was true for allergies and atopic diseases for a long time. The assertions by Langen et al.^7^, that the prevalence rates for allergy are lower in former East Germany than in West Germany can only be partially supported in this study: Although the prevalence rates of AA and ARC are significantly lower in eastern Germany, we see higher prevalence rates of AD in east Germany.

It should be noted that differences in morbidity (here, prevalence rates) between eastern and western Germany vary by age. These differences are even more pronounced among older people and can be attributed to the different characteristics of the determinants of health (including the healthcare system and health behavior) in the former Federal Republic of Germany and the German Democratic Republic. This is evident, for example, in the use of early detection and preventive services. A striking example is the willingness to be vaccinated, which is significantly higher among older people in eastern than in western Germany (e.g., Riens et al. 2013^23^). These differences are not found among younger people (Goffrier et al. 2016^24^). It is therefore evident that a certain degree of convergence has taken place, including in terms of health behavior, and that the differences between the East and the West are no longer quite as pronounced. The convergence of living conditions in eastern and western German counties since reunification is also reflected in the fact that certain skin diseases, particularly those that occur in childhood, no longer exhibit such significant regional differences between the two regions.^7^ However, this should not obscure the fact that there are still numerous regional variations in morbidity, mortality, and health behavior.

Regional socio‐demographic and environmental factors may also have an important influence on the prevalence rates of atopic diseases.^1^, ^25^ Especially with regard to the correlations between AD and AA, as well as between AA and ARC, influences of these external factors need to be investigated in more detail.

A major strength of this study is the large dataset, which covers approximately 90% of the German population and almost all individuals insured under statutory health insurance. It provides the opportunity to analyze all three diseases over a nine‐year period across all 401 German counties, allowing trends and characteristics over nearly a decade to be examined. Through various statistical and geographical analyses, the regional differences and correlations between these diseases were identified.

Although there are many studies on the spread or on comorbidities of the individual diseases, all three diseases have only rarely been considered and analyzed simultaneously, to the same extent. This study makes an important contribution to the improvement of care and knowledge of the atopic march.

A limiting factor is that the data on the number of insured persons was only available for 2020 and therefore had to be estimated for the other study years. However, with this large dataset, we can assume that the resulting inaccuracies are rather small. It should also be noted that the dataset is based on the billing frequencies of outpatient physicians. The prevalence, therefore, excludes patients who did not consult a physician in the respective year and could also include misdiagnosed patients. Attention must also be drawn to a possible ecological fallacy: As person‐level data are not available, regional associations do not necessarily indicate that the same individuals have several atopic diseases at the same time.

## CONCLUSIONS

In this study, the regional prevalence rates of atopic dermatitis, allergic asthma, and rhinoconjunctivitis in Germany were investigated. Increasing prevalence rates over the past decade, and a north‐south gradient and a west‐east gradient were identified.

Future investigations should focus on identifying potential reasons for these findings and include further areas, such as socio‐economic, geographical, and political factors.

## CONFLICT OF INTEREST STATEMENT

None.

## Supporting information



Supplementary information
