# Supplementary figures and images for: Regional variations and temporal trends of atopic diseases in Germany
Source: J Dtsch Dermatol Ges. 2026 Jan 16;24(5):634–41. doi: 10.1111/ddg.15926 (PMC13140137; doi:10.1111/ddg.15926)

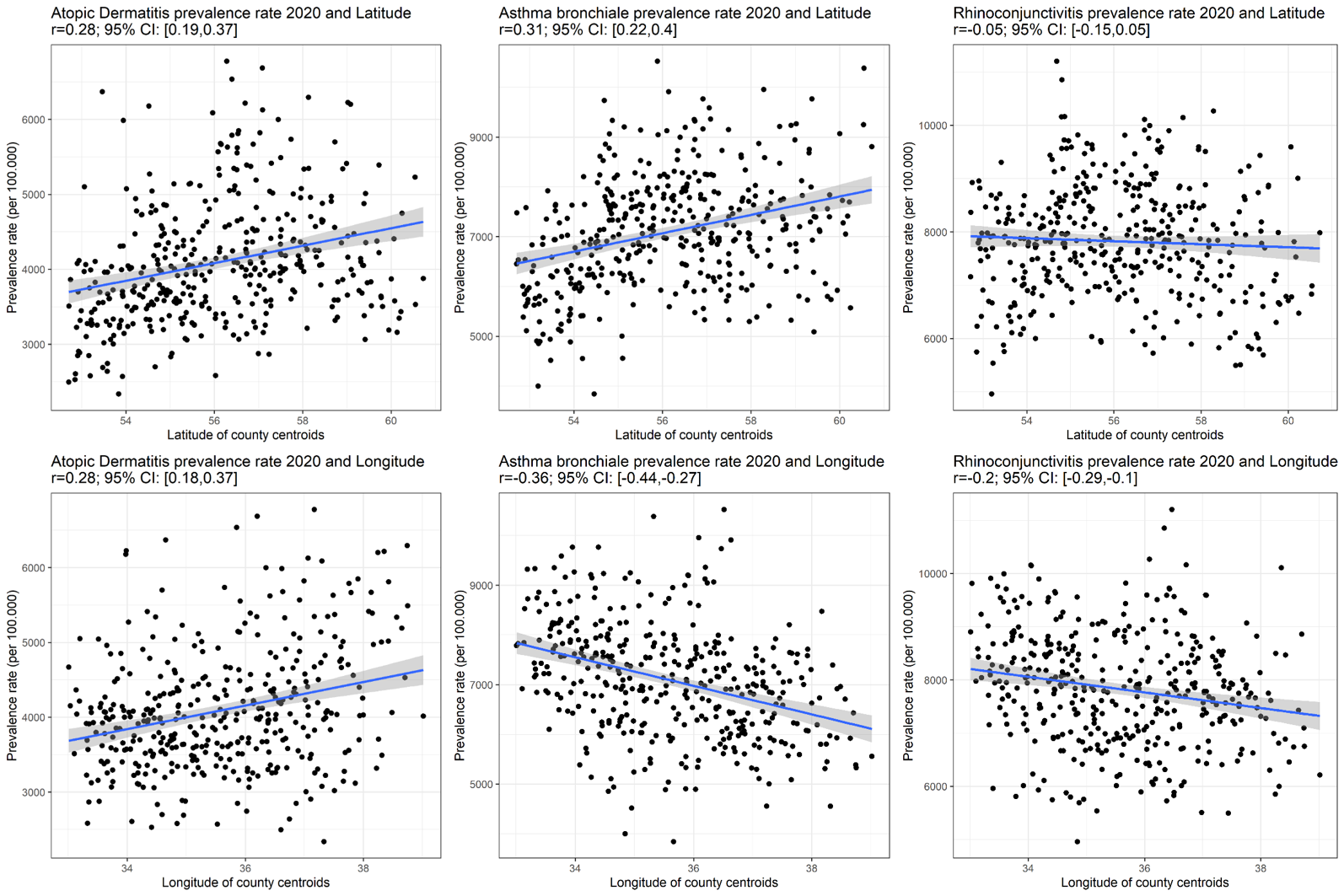

Supplement: Supplementary file 1 — Supplementary information [file DDG-24-634-s001.tif]
